# Association between small-for-gestational-age at birth and placental abruption in normotensive pregnancies: a retrospective cohort study

**DOI:** 10.1186/s12884-026-09156-4

**Published:** 2026-04-30

**Authors:** Qingwen Nie, Yao Xu, Zixian Wan, Fan Hong, Chi Chiu Wang, Zhijian Wang

**Affiliations:** 1 Department of Obstetrics and Gynecology, Guangdong Provincial Key Laboratory of Major Obstetric Diseases, Guangdong Provincial Clinical Research Center for Obstetrics and Gynecology, Guangdong-Hong Kong-Macao Greater Bay Area Higher Education Joint Laboratory of Maternal-Fetal Medicine, The Third Affiliated Hospital, Guangzhou Medical University, No. 63 Duobao Road, Liwan District, Guangzhou, Guangdong 510150 China; 2 Department of Obstetrics and Gynaecology, Prince of Wales Hospital, The Chinese University of Hong Kong, Shatin, Hong Kong SAR, China; 3 Center for Reproductive Medicine, The Third Affiliated Hospital, Guangzhou Medical University, Guangzhou, 510150 China

**Keywords:** Normotensive pregnancies, Placental abruption, Impaired fetal growth, Ischemic placental disease, Placental aging

## Abstract

**Background:**

Hypertensive disorders in pregnancy (HDP) are well-established risk factors for placental abruption (PA). Impaired fetal growth is another candidate, yet its association with abruption in the absence of hypertension remains unverified. We used small-for-gestational-age (SGA) at birth as a pragmatic proxy to investigate whether it is independently associated with PA in normotensive pregnancies.

**Methods:**

A retrospective cohort study was conducted at a tertiary hospital in South China. The study population consisted of women with singleton deliveries between January 1, 2019, and December 31, 2024. Fetal growth was categorized using sex-specific percentiles of birthweight for gestational age. Birthweight < 10th percentile reflects SGA at birth. Severe SGA refers to < the 3rd percentile (P3), and those > P10 was the reference group.

**Results:**

A review of 36,544 singleton deliveries identified 654 cases of PA, of which 521 (80%) occurred in normotensive pregnancies (*n* = 32,269). SGA at birth was significantly associated with a higher odds of PA (2.2% vs. 1.5%, adjusted OR 1.6, 95% CI 1.21–2.16). Other associated factors for abruption included age ≥ 35 years, nulliparity, anemia, and preterm gestation. Subgroup analyses based on these factors showed that the < P3 cohort had significantly greater risk of abruption than the> P10 cohort. The corresponding relative risks (RRs) were 2.3, 1.7, 1.7, and 2.3 in the ≥ 35 years, nulliparity, anemia, and term gestation, respectively. In the comparison of the P3-P10 and the > P10 cohorts, the RRs were both 1.8 in the ≥ 35 years and term gestation. The risk between < P3 and P3–P10 was not significant in any subgroup.

**Conclusions:**

These findings suggest that SGA at birth is independently associated with an elevated risk of PA in normotensive pregnancies, potentially explained by a shared pathophysiology of ischemic placental disease. Increased vigilance is warranted, particularly in SGA pregnancies complicated by advanced maternal age and term gestation.

**Supplementary Information:**

The online version contains supplementary material available at 10.1186/s12884-026-09156-4.

## Background

Placental abruption (PA) is defined as the partial or complete separation of a normally located placenta from the uterine wall prior to delivery, exclusively in pregnancies beyond 20 weeks. It affects approximately 1% of pregnancies, and its incidence has increased in recent decades [1, 2]. PA often requires prompt delivery and is associated with substantial perinatal morbidity and mortality [3]. Population-based studies have indicated that perinatal mortality in PA cases is 15–20 times greater than that in pregnancies without abruption, with the majority of deaths occurring prior to birth [4–6]. Among live-born offspring, exposure to PA was associated with a sixfold increase in the risk of cerebral palsy [7].

The causes of PA are heterogeneous and remain largely speculative. Hypertensive disorders during pregnancy (HDP), particularly preeclampsia with severe features, are strong risk factors for abruption [2, 3]. While prior studies reported an association between fetal growth restriction and PA, their findings often overlooked the mediating effect of HDP [1, 2]. The precise magnitude of this association, specifically in normotensive pregnancies, where the isolated effect of placental insufficiency might be discerned, remains less clear. Clarifying this relationship may offer important insights into the pathogenesis of PA. Given the retrospective design and the absence of antenatal Doppler assessments, we used small-for-gestational-age (SGA) at birth as a proxy for impaired fetal growth. This study systematically evaluates the relationship between SGA at birth and PA risk in normotensive pregnancies. Given that severity of growth restriction may reflect underlying placental insufficiency, we examined the associations separately using the 10th and 3rd percentile cutoffs.

## Methods

### Study design

This was a retrospective cohort study conducted at the Third Affiliated Hospital of Guangzhou Medical University, a tertiary teaching hospital in South China. Singleton deliveries between January 1, 2019, and December 31, 2024, were retrieved from the institutional perinatal database. The study protocol was approved by the institutional review board (IRB-2025-083). All women who delivered during this period signed a general informed consent form upon admission. Owing to its retrospective nature, specific written informed consent was waived. This study was conducted in accordance with the Declaration of Helsinki.

Inclusion Criteria: (1) Singleton pregnancies with delivery records. Exclusion Criteria: (1) Pregnancies delivering before 24 weeks or after 42 weeks of gestation; (2) Pregnancies with missing data on newborn’s sex or weight. (3) Pregnancies complicated by HDP. HDP categories included chronic hypertension, gestational hypertension, preeclampsia-eclampsia, and chronic hypertension with superimposed preeclampsia [8]. Normotensive pregnancies (non-HDP) were selected and stratified according to the presence or absence of SGA at birth. The primary outcome was the incidence of PA.

### Data collection and definitions

Maternal age, body mass index (BMI), mode of conception, gravidity, parity, and pregnancy complications, such as hyperglycemia in pregnancy, anemia, polyhydramnios, oligohydramnios, prelabor rupture of membranes, and chorioamnionitis, were documented via chart review. Hyperglycemia in pregnancy included both pre-gestational diabetes mellitus (PGDM) and gestational diabetes mellitus (GDM). Detailed definitions of the variables are summarized in supplementary table. Gestational age (GA) at delivery, mode of delivery, neonatal birth weight, Apgar score at 1 min and 5 min were collected manually from medical records. Pregnancy outcomes including placental abruption, postpartum hemorrhage, hysterectomy, hypovolemic shock, disseminated intravascular coagulation (DIC), maternal intensive care unit (ICU) and neonatal intensive care unit (NICU) admission, fetal distress and stillbirth were identified according to diagnostic codes. GA was routinely calibrated at the time of the nuchal translucency scan (11–13^+ 6^ weeks) based on crown–rump length measurements. Fetal growth was assessed using ultrasound by monitoring estimated fetal weight (EFW) and identifying growth deviations from normal patterns [9]. The measurement of EFW was based on fetal biometric parameters, including head circumference, abdominal circumference, and femur length [10]. SGA at birth referred to birthweight < 10th percentiles for gestational age, which served as a pragmatic proxy for impaired fetal growth in this study. In accordance with Chinese neonatal birthweight between 24 and 42 weeks of gestation, distinct criteria are applied for male and female infants [11]. Subgroups were created to assess the risk of PA according to the severity of SGA: < P3, P3–P10, and ≥P10 (reference group). Classic clinical signs of PA included painful vaginal bleeding, uterine hypertonicity, vaginal bleeding, and/or nonreassuring fetal status. Placental abruption diagnosis was confirmed either by (1) visualization of a retroplacental clot upon delivery of the placenta or (2) prenatal ultrasonographic findings of placental separation. All diagnoses were made by certified obstetricians and recorded in the medical record system. The condition is graded from 0 to 3 on the basis of clinical severity [12]. Grade 0 refers to small retroplacental clots that are asymptomatic, with the diagnosis established based on visual examination of the placenta. Grade 1 is defined as no or scant vaginal bleeding, mild uterine tenderness, and no maternal shock or fetal distress. Grade 2 is defined as no to moderate vaginal bleeding, a hypertonic uterus with marked tenderness, and fetal distress without maternal shock. Grade 3 placental abruption refers to profuse vaginal bleeding, a rigid ‘board-like’ uterus, maternal shock, stillbirth, and possibly coagulopathy. Grades 0 ~ 1 represents concealed abruption, whereas grades 2 ~ 3 represents revealed abruption.

### Statistical analysis

Statistical analyses were performed via SPSS software, version 22.0 (IBM Corp., Armonk, NY, USA). The Mann–Whitney U test was applied for continuous variables (presented as medians [interquartile ranges]), and the chi-square test was used for categorical variables (presented as n [%]). Maternal characteristics were compared between SGA group and non-SGA group. The variables with missingness were maternal BMI and gestational weight gain, each with a missing proportion of 14.6%. Multiple imputation by chained equations (MICE) was performed using SPSS. All variables in the multivariable model were included as auxiliary variables for the imputation. Covariates included in the multivariable model were selected a priori based on clinical relevance and prior literature, including maternal age, parity, gestational diabetes or preexisting diabetes, polyhydramnios, chorioamnionitis, etc. In our multivariable logistic regression model, all covariates entered simultaneously. There were 521 placental abruption events and 15 covariates, yielding an events per variable (EPV) of 34.7, indicating that the risk of overfitting is very low and that the model estimates are stable. A forest plot was constructed to visualize the adjusted odds ratio (OR) and 95% confidence interval (CI). Then, we assessed the relative risk (RR) of PA associated with different severity of SGA (< P3 vs. P3-P10 vs. ≥P10). Finally, independent factors for PA among normotensive women with SGA were evaluated. The results with *P* < 0.05 were regarded as statistically significant.

## Results

**Fig. 1:**
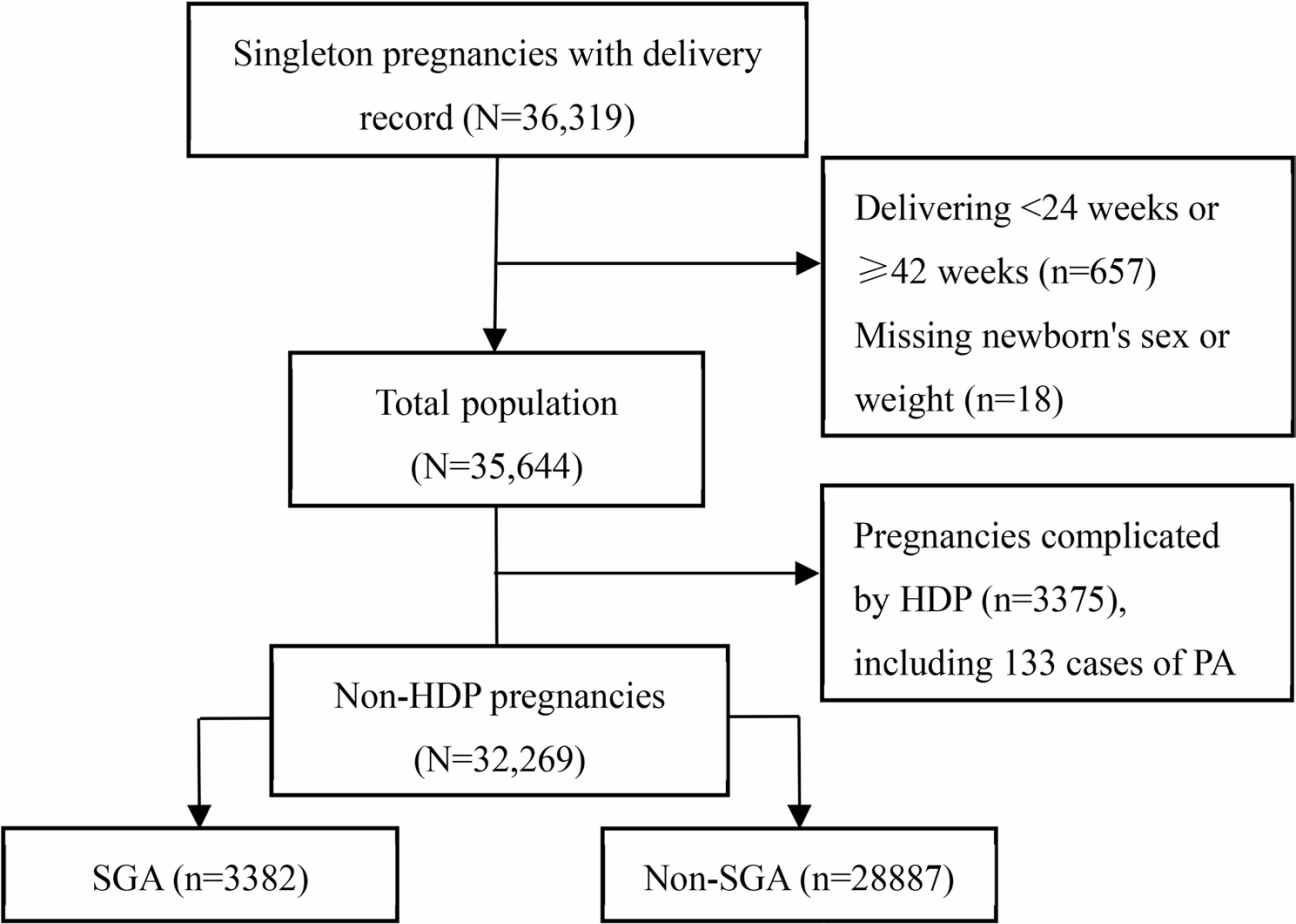
Flow diagram of the study population selection and stratification

A total of 36,319 singleton pregnancies with complete delivery records were initially identified. The selection process of the study population is illustrated in Fig. 1. Pregnancies with delivery before 24 weeks or at or beyond 42 weeks of gestation, and those with missing data on neonatal sex or birth weight were excluded. Subsequently, 3,375 pregnancies complicated by HDP were excluded, with PA occurring in 133 cases (3.9%). The final study cohort consisted of 32,269 normotensive (non-HDP) pregnancies, including 3,382 pregnancies with SGA at birth (10.5%) and 28,887 pregnancies without SGA (89.5%).

Among the normotensive pregnancies with or without SGA, significant differences were observed between the groups, as shown in Table 1. Women in the SGA group were younger (median age 30.0 vs. 31.0 years, *p* < 0.001), had a lower baseline BMI (21.1 vs. 22.3 kg/m², *p* < 0.001), and gained less weight during pregnancy (median 9.5 kg vs. 10.5 kg, *p* < 0.001). The ART use rate among women with and without SGA were 11.6% and 14.5%, respectively (*p* < 0.001). The proportion of nulliparous women was also markedly greater in the SGA group (67.5% vs. 50.8%, *p* < 0.001). Pregnancies complicated by SGA were associated with a significantly higher prevalence of congenital uterine anomalies (1.2% vs. 0.8%, *p* = 0.013) and autoimmune diseases (3.1% vs. 2.1%, *p* < 0.001) compared to non-SGA pregnancies. In terms of pregnancy complications, the rates of hyperglycemia in pregnancy and anemia were significantly lower in the SGA group (15.5% vs. 20.5%, *p* < 0.001; 15.1% vs. 17.3%, *p* = 0.001), whereas oligohydramnios and oxytocin induction were more common (16.8% vs. 7.8%, 32.8% vs. 28.7%, both *p* < 0.001). In contrast, polyhydramnios, prelabor rupture of membranes, and chorioamnionitis were less common in the SGA group.

**Table 1:** Maternal characteristics of normotensive pregnancies with and without SGA

	SGA (*n* = 3382)	Non-SGA (*n* = 28887)	*P* value
Maternal age, year	30.0 (27.0, 34.0)	31.0 (28.0, 35.0)	<0.001*
<35 years	2717 (80.3)	21,233 (73.5)	<0.001*
≥ 35 years	665 (19.7)	7654 (26.5)	
Baseline BMI, kg/m^2^	21.3 (19.5, 23.5)	22.4 (20.4, 24.8)	<0.001*
Gestational weight gain, kg	9.6 (6.9, 12.0)	9.8 (6.7, 12.5)	0.047*
Mode of conception			<0.001*
Natural	2989 (88.4)	24,684 (85.5)	
ART	393 (11.6)	4203 (14.5)	
Parity status			<0.001*
Nulliparous	2284 (67.5)	14,674 (50.8)	
Multiparous	1098 (32.5)	14,213 (49.2)	
Congenital uterine anomalies	42 (1.2)	238 (0.8)	0.013*
Autoimmune diseases	104 (3.1)	596 (2.1)	<0.001*
Hyperglycemia in pregnancy	523 (15.5)	5913 (20.5)	<0.001*
Anemia	509 (15.1)	5003 (17.3)	0.001*
Hyperthyroidism	47 (1.4)	340 (1.2)	0.282
Hypothyroidism	118 (3.5)	1180 (4.1)	0.095
Polyhydramnios	10 (0.3)	274 (0.9)	<0.001*
Oligohydramnios	567 (16.8)	2246 (7.8)	<0.001*
Prelabor rupture of membranes	658 (19.5)	6269 (21.7)	0.003*
Oxytocin induction	1108 (32.8)	8277 (28.7)	<0.001*
Chorioamnionitis	28 (0.8)	618 (2.1)	<0.001*

*SGA* small for gestational age, *BMI* body mass index, *ART* assisted reproductive technology, *SLE* systemic lupus erythematosus

*P* values for categorical variables were calculated using the chi-square test, and continuous variables were calculated using the Mann‒Whitney U test

*Results presented in bold are statistically significant

**Fig. 2:**
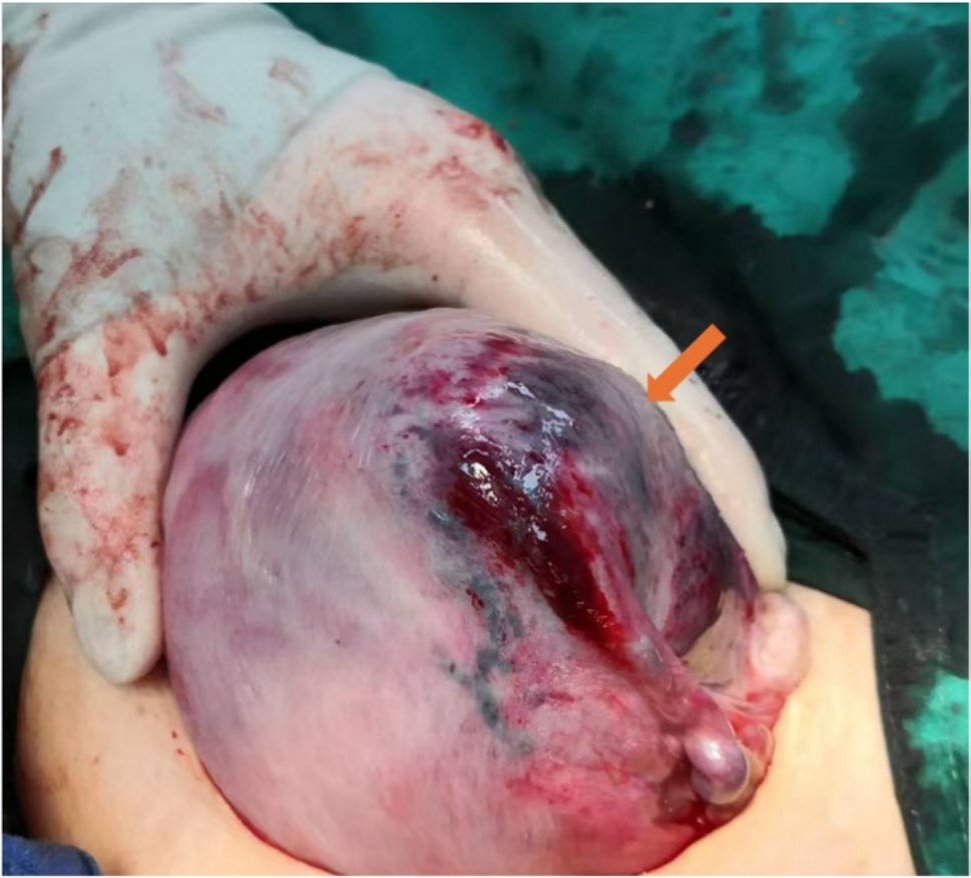
Couvelaire uterus following severe placental abruption. Intraoperative appearance of a Couvelaire uterus showing diffuse bluish-purple discoloration of the uterine serosa (arrows), caused by extravasation of blood into the myometrium following severe placental abruption

Table 2 presents a comparison of pregnancy outcomes between the two groups. The distribution of GA at delivery differed significantly, with a greater proportion of term gestation in the SGA group (91.2% vs. 87.9%, *p* < 0.001). Placental abruption occurred in 74 pregnancies (2.2%) in the SGA group and 447 pregnancies (1.5%) in the non-SGA group (*p* = 0.005). Additionally, revealed abruption (Grade 2–3) were more frequent in the SGA group than in the non- SGA group (44.6% vs. 30.4%, *p* = 0.016). The presence of a cafolaire uterus was a manifestation of severe placental abruption (Fig. 2). Cesarean delivery and postpartum hemorrhage occurred less commonly in the SGA group (35.4% vs. 40.4% and 5.6% vs. 10.1%, both *p* < 0.001). The incidence of hysterectomy was also reduced in the SGA group (0.2% vs. 0.7%, *p* = 0.001). However, the SGA group presented significantly higher rates of fetal distress and stillbirth (17.5% vs. 9.6% and 3.1% vs. 1.8%, both *p* < 0.001). Low birth weight was more common in the SGA group than in the non- SGA group (25.8% vs. 6.8%, *p* < 0.001), while the NICU admission rate was comparable between the two groups (16.4% vs. 16.6%, *p* = 0.771). There were no significant differences in the neonates with a 1-minute or 5-minute Apgar score below 7.

**Table 2:** Pregnancy outcomes between normotensive pregnancies with and without SGA

	SGA (*n* = 3382)	Non-SGA (*n* = 28887)	*P* value
Term gestation	3084 (91.2)	25,384 (87.9)	<0.001*
Placental abruption	74 (2.2)	447 (1.5)	0.005*
Degree 0 ~ 1	41 (55.4)	311 (69.6)	0.016*
Degree 2 ~ 3	33 (44.6)	136 (30.4)	
Mode of delivery			<0.001*
Vaginal	2185 (64.6)	17,217 (59.6)	
Cesarean	1197 (35.4)	11,670 (40.4)	
Postpartum hemorrhage	188 (5.6)	2927 (10.1)	<0.001*
Hypovolemic shock	6 (0.2)	65 (0.2)	0.576
DIC	2 (0.1)	12 (0.0)	0.642
Hysterectomy	7 (0.2)	201 (0.7)	0.001*
ICU admission	27 (0.8)	233 (0.8)	0.960
Fetal distress	591 (17.5)	2767 (9.6)	<0.001*
Stillbirth	104 (3.1)	524 (1.8)	<0.001*
1 min Apgar score < 7	75 (2.3)	745 (2.6)	0.248
5 min Apgar score < 7	6 (0.2)	82 (0.3)	0.275
LBW infant ^a^	846 (25.8)	1933 (6.8)	<0.001*
NICU admission ^a^	537 (16.4)	4703 (16.6)	0.771

*SGA* small for gestational age, *DIC *disseminated intravascular coagulation, *ICU *intensive care unit, *Apgar *appearance, pulse, grimace, activity, respiration, *LBW *low birth weight, *NICU *neonatal intensive care unit

*P* values for categorical variables were calculated using the chi-square test. *Results presented in bold are statistically significant

^a^ Data were calculated using the number of live births in each group as the denominator (n = 3,278 for the SGA group and n = 28,363 for the non-SGA group)

**Fig. 3:**
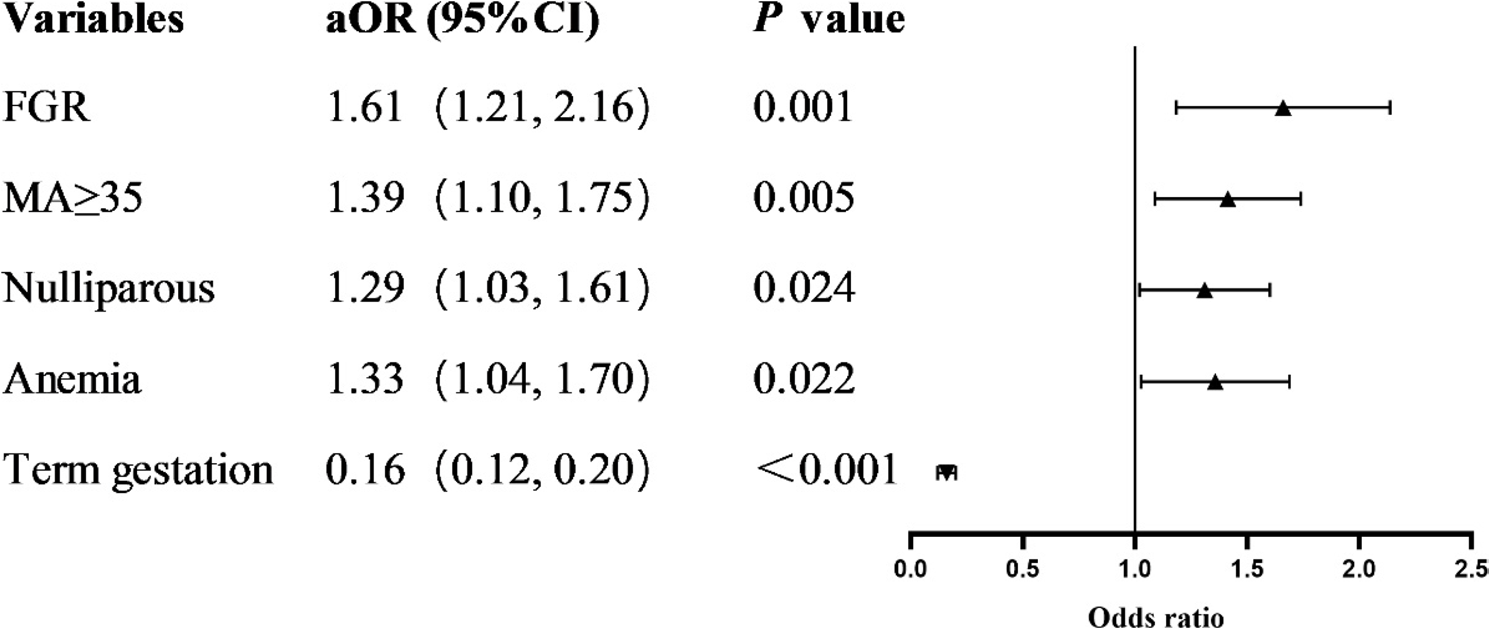
Multivariable logistic regression for risk factors associated with placental abruption. Forest plot showing adjusted odds ratios (ORs) and 95% confidence intervals (CIs) for the association between risk factors and placental abruption in a multivariable logistic regression analysis

We performed a multivariate logistic regression, as shown in Fig. 3, to assess the risk factors for PA. In normotensive pregnancies, SGA is associated with an increased risk of abruption by 61% compared with its absence (aOR, 1.61; 95% CI, 1.21–2.16; *p* = 0.001). Advanced maternal age was independently associated with a 39% higher risk of placental abruption, even though it was less common in the SGA group (19.7% vs. 26.5%, *p* < 0.001). Nulliparity and anemia were associated with 29% and 33% elevated risks of abruption, respectively. Notably, term gestation was identified as a protective factor against abruption (aOR, 0.16; 95% CI, 0.12–0.20), indicating a significantly reduced risk compared with preterm gestation. After adjustment, other factors did not show a statistically significant association. In a sensitivity analysis restricted to clinically revealed placental abruption (Grades 2–3), the association with SGA at birth remained statistically significant (adjusted OR 1.92, 95% CI: 1.22–3.02).

Stratified analyses were performed to assess the risk of abruption by different degrees of SGA among normotensive pregnancies, as detailed in Table 3. The incidence of abruption was consistently greater in the < P3 group than in both the P3–P10 and ≥P10 groups. Among women ≥ 35 years, the < P3 group had a 2.3-fold increased risk of abruption (RR 2.3, 95% CI 1.1–4.8) compared with the ≥P10 group. The association between SGA and placental abruption differs in preterm versus term pregnancies, with the risk being more pronounced at term (RR 2.3, 95% CI 1.4–3.7). Nulliparous women in the < P3 group presented a 1.7-fold increased risk, and a comparable RR was present among women without anemia. In the comparison of P3–P10 versus ≥P10, the association was only significant among advanced maternal age and term gestation subgroups (both 1.8-fold risk). However, the relative risk of < P3 versus P3–P10 did not reach statistical significance in any subgroup. In the subgroups of women with advanced maternal age, who were parous, had anemia, or delivered preterm, the absolute number of abruption events in < P3 category was small (*n* = 5 ~ 7), resulting in wide CIs of RR_12_ and RR_13_.

**Table 3:** Risk of placental abruption by SGA in subgroups among normotensive pregnancies

	< P3 *n* (%)	P3-P10 *n* (%)	≥P10 *n* (%)	RR_12_ (95%CI)	RR_13_ (95%CI)	RR_23_ (95%CI)
Subgroup 1						
MA<35	16 (2.3)	34 (1.7)	303 (1.4)	1.4 (0.8–2.5)	1.6 (1.0-2.7)	1.2 (0.8–1.7)
MA ≥ 35	7 (4.3)	17 (3.4)	144 (1.9)	1.3 (0.5-3.0)	**2.3 (1.1–4.8)**	**1.8 (1.1–2.9)**
Subgroup 2						
Nulliparous	17 (2.8)	35 (2.1)	243 (1.7)	1.4 (0.8–2.4)	**1.7 (1.1–2.8)**	1.3 (0.9–1.8)
Parous	6 (2.4)	16 (1.9)	204 (1.4)	1.3 (0.5–3.3)	1.7 (0.8–3.8)	1.3 (0.8–2.2)
Subgroup 3						
Anemia	5 (3.8)	10 (2.6)	97 (1.9)	1.4 (0.5–4.1)	2.0 (0.8–4.8)	1.4 (0.7–2.6)
Normal	18 (2.5)	41 (1.9)	350 (1.5)	1.3 (0.8–2.3)	**1.7 (1.1–2.7)**	1.3 (0.9–1.8)
Subgroup 4						
Preterm	6 (5.9)	10 (5.1)	197 (5.6)	1.2 (0.4–3.1)	1.1 (0.5–2.3)	0.9 (0.5–1.7)
Term	17 (2.3)	41 (1.8)	250 (1.0)	1.3 (0.7–2.3)	**2.3 (1.4–3.7)**	**1.8 (1.3–2.5)**

*SGA *small for gestational age, *MA *maternal age. The data are presented as n (incidence, %) and relative risk (RR) values with 95% confidence intervals (CIs). RR_12_: relative risk of 13: relative risk of 23: relative risk of P3–P10 vs. ≥P10 (reference)

The results presented in bold are statistically significant, as their 95% CIs do not include the value of 1.0

We further analyzed risk factors associated with abruption specifically within the SGA population. As presented in Table 4, advanced maternal age (≥ 35 years) and preterm gestation were identified as independent risk factors for PA. After adjusting for covariates, women of advanced age had 1.81-fold increased odds of PA, while those with preterm gestation had 2.74-fold increased odds. Hyperglycemia in pregnancy was associated with PA in univariate analysis but was not statistically significant in the multivariate model.

**Table 4:** Risk factors associated with PA among normotensive women with SGA

Variables (reference group)	Univariate analysis OR (95% CI)	Multivariate Logistic aOR (95% CI)
Advanced maternal age (≥ 35 vs. <35 years)	**1.96 (1.21–3.17)**	**1.81 (1.10–2.99)**
Hyperglycemia in pregnancy (yes vs. no)	**1.76 (1.04–2.96)**	1.54 (0.89–2.67)
Preterm gestation (< 37 vs. ≥37 weeks)	**2.86 (1.66–4.90)**	**2.74 (1.55–4.85)**

*PA* placental abruption, *SGA* small for gestational age, *aOR* adjusted odds ratio, *CI* confidence interval. The results presented in bold are statistically significant, as their 95% CIs do not include the value of 1.0

## Discussion

This retrospective cohort study demonstrates an independent association between SGA at birth and placental abruption in normotensive pregnancies. Moreover, this association is amplified in the context of birthweight below the 3rd percentile, maternal age ≥ 35, and term deliveries. It should be noted that SGA at birth represents a heterogeneous group that includes both constitutionally small but healthy newborns and true pathological growth restriction. This misclassification bias likely dilutes the true association between placental dysfunction and abruption, meaning our effect estimates may be conservative. Moreover, the findings of this study should be interpreted as an association rather than a causal relationship. Reverse causality (i.e., placental abruption leading to SGA) and shared underlying placental pathology cannot be ruled out. Future studies with antenatal ultrasound data are needed to establish temporality and causality.

A large population-based study in Japan reported the highest risk of abruption among women with severe preeclampsia, although no significant differences were observed among HDP phenotypes [13]. Therefore, the present study regarded HDP as a unified category to filter the study population. Cohort studies indicated that abruptions complicated by preeclampsia and/or intrauterine growth restriction are associated with worse maternal and neonatal outcomes [14, 15]. Evidence also consistently demonstrated that impaired fetal growth may act as a modifier in the association between exposure factors and abruption [16, 17]. A study involving women with chronic hypertension reported an inverse dose‒response relationship between the incidence of PA and neonatal birthweight percentiles, which was particularly evident below the 3rd percentile [16]. Similarly, the association between major congenital anomalies and abruption was markedly amplified among births complicated by SGA at birth [17]. However, our analysis shows that nearly 80% of abruptions occur in non-HDP populations. Given that more than half of PAs in normotensive pregnancies are grade 0–1 with no or subtle clinical signs, identifying them early remains challenging. Estimated fetal weight falling below the expected percentile for gestational age can be regarded as an early warning indicator for PA. This study used SGA at birth as a pragmatic proxy for impaired fetal growth. Emerging evidence showed that Doppler ultrasound detecting abnormal umbilical artery waveforms may signal placental deterioration [18]. However, Doppler indexes applied in current practice show insufficient capacity to reliably differentiate between the two entities [19].

It is important to note that the SGA pregnancies were associated with a higher likelihood of Grade 2–3 abruption, which reflect severe placental abnormalities. A key pathophysiology of impaired fetal growth is placental ischemia, which may result from the inadequate trophoblast invasion and abnormal uteroplacental vascular development. This highlights the importance of monitoring pregnancies with SGA fetus. Our findings reinforce the conceptual framework of ischemic placental disease, in which preeclampsia, fetal growth restriction, and abruption are different clinical phenotypes of a shared pathophysiological process [20, 21]. A deeper understanding of PA includes vascular inflammation, inadequate trophoblastic invasion, and preterm placental aging [22–26]. However, in contrast to the systemic nature of HDP-induced impaired fetal growth, those in normotensive pregnancies seems more likely to represent a placenta-confined disorder [27, 28]. Atsumi et al. demonstrated evidence of localized placental oxidative stress rather than systemic oxidative stress and maternal endothelial dysfunction [28]. Additionally, the pathogenic mechanisms differ between early-onset and late-onset subtypes: the former is associated primarily with maternal placental hypoperfusion, whereas the latter additionally involves fetal vascular pathology [29]. A recent systematic review indicated that elevated serum sFlt-1 levels and increased sFlt-1/PlGF ratios correlate with the risk of placental abruption but show no discriminatory value in pregnant women with preeclampsia [30]. We speculate that sFlt-1 or the sFlt-1/PlGF ratio may serve as promising auxiliary indicators for identifying normotensive pregnancies at risk of PA, which needs further exploration.

Our finding that SGA is associated with an increased risk of PA raises important clinical considerations. The intervention of delivery in pregnancies complicated by SGA requires careful deliberation. Although our study did not demonstrate a significant association between induction and abruption, tachysystole is clinically believed to increase the risk of placental abruption. Obstetricians should balance the risks of ongoing placental insufficiency against the potential iatrogenic risks of induction. The findings are consistent with a previous report that approximately half of abruption cases occur before term gestation [2]. The etiologic factors underlying PA generally differ between term and preterm pregnancies [22]. The finding that term gestation is a protective factor for placental abruption overall does not conflict with the observation that the SGA-abruption association is stronger at term. A plausible interpretation is that among term pregnancies, SGA identifies a subgroup with underlying chronic placental insufficiency that remains vulnerable to abruption. In contrast, preterm placental abruption is more often driven by acute processes (e.g., inflammation, vascular events) that may occur independently of fetal growth status. Alternatively, residual confounding or statistical artifacts due to small sample sizes in subgroup analyses cannot be excluded. Generally, preterm abruption is more frequently associated with severe maternal–fetal complications [31]. The association between PA and low birthweight is well established and is primarily mediated through preterm birth rather than impaired fetal growth [32]. Additionally, preterm delivery acts as the crucial mediator of abruption-related perinatal mortality, and the effect is greater at earlier gestational ages [4]. In clinical practice, expectant management is often pursued for isolated growth-restricted fetus to prolong gestation to term [10]. However, our findings suggest that the risk of PA associated with SGA at birth is more pronounced when pregnancies reach full term. One possible explanation is that placental aging leads to structural instability, thereby increasing the risk of PA [23]. In preterm delivery, the causes of abruption are often multifactorial, which could obscure the specific contribution of SGA [22]. The higher rates of preterm delivery, postpartum hemorrhage and hysterectomy observed in the non-SGA group compared to the SGA group should be interpreted with caution. The non-SGA group had a higher prevalence of known risk factors for these outcomes, including advanced maternal age, gestational diabetes mellitus, and assisted reproductive technology use, which may partially explain the observed differences rather than any protective effect of SGA. Advanced maternal age and primiparity are recognized as common risk factors for abruption [2]. Maternal age above 40 years was associated with an increased risk of PA, potentially mediated by reduced vascular compliance and endothelial dysfunction [33, 34]. Similarly, women with parity ≥ 3 may significantly elevate the risk of abruption by 60% [34]. These two factors may synergize in creating a milieu more prone to vessel rupture and abruption. However, established evidence showed that ischemic placental diseases are more prevalent in nulliparous women because of maternal cardiac maladaptation [35, 36]. While speculative, these hypotheses align with known physiology and warrant investigation in future studies. Anemia is an identifiable etiologic factor for both impaired fetal growth and abruption [37]. The elevated baseline risk of abruption in anemic pregnancies may mask the effect of SGA. Notably, the subgroups of severe SGA with a limited sample size compromised the reliability of these estimates. Therefore, these findings are presented as exploratory and hypothesis-generating rather than definitive, and the results should be interpreted with caution.

### Strengths and limitations

A key strength of this study lies in revealing a direct association between SGA at birth and the occurrence of PA. The exclusion of hypertensive disorders in our cohort ensures a more isolated assessment. This association also holds true for women with hypertension, or stronger, as confirmed by previous studies. The large sample size and single-center design together ensure both statistical robustness and internal consistency. Our study employed robust statistical approaches, including multivariable logistic regression models with adjustment for potential confounders. The stratified analyses further allow risk differentiation across various clinical situations and confirm the robustness of the results. However, several limitations should be disclosed. First, we explicitly acknowledge the inherent limitation of using SGA at birth as a surrogate for placental dysfunction. Our findings actually reflect the association between birthweight below the 10th percentile and placental abruption risk, which may differ from the risk profile of true pathological growth restriction. The second limitation is our retrospective study design, including the potential for confounding by indication, missing variable bias, and misclassification bias. Certain confounding factors (such as smoking, egg donation, prior preeclampsia or thrombophilias, and placental abruption area) were unavailable in the dataset due to sparse data resulting from low event counts and inadequate documentation, despite the use of standardized medical records. Additionally, the timing of placental abruption relative to the onset of impaired fetal growth was difficult to determine for the lack of serial antenatal ultrasound data. Therefore, this study identifies important associations but cannot establish causality. Given that some subgroup analyses may be underpowered due to small sample size, the results should be interpreted with caution. Formal interaction tests should be conducted if the sample size provides sufficient statistical power. Finally, placental pathology could elucidate abnormalities underlying placental abruption, an area warranting future research.

## Conclusions

In this retrospective cohort, SGA at birth was associated with increased odds of PA in normotensive pregnancies. These findings likely reflected a shared underlying placental pathology between SGA and PA, consistent with an associational rather than a causal relationship. From a clinical perspective, pregnancies with SGA fetus warrant increased vigilance for symptoms that could overlap with PA, such as unexplained abdominal pain, vaginal bleeding, or fetal heart rate decelerations. Intensive fetal growth assessments and evaluation of abnormal signs may be beneficial in pregnancies complicated by SGA. Although these exploratory findings require confirmation, women of advanced maternal age and those at term may prompt heightened awareness.

## Supplementary Information

Supplementary Material 1.

## Data Availability

The minimal dataset necessary to reproduce the findings reported in this manuscript is available from the corresponding author upon reasonable request. The full datasets are not publicly available, as they contain information that is part of an ongoing study.

## Abbreviations

ART: Assisted reproductive technology
BMI: Body mass index
CI: Confidence interval
DIC: Disseminated intravascular coagulation
SGA: Small for gestational age
GA: Gestational age
HDP: Hypertensive disorders during pregnancy
ICU: Intensive care unit
NICU: Neonatal intensive care unit
OR: Odds ratio
PA: Placental abruption
RR: Relative risk
