# Supplementary material for: Association between small-for-gestational-age at birth and placental abruption in normotensive pregnancies: a retrospective cohort study
Source: BMC Pregnancy Childbirth. 2026 Apr 30;26:660. doi: 10.1186/s12884-026-09156-4 (PMC13277014; doi:10.1186/s12884-026-09156-4)
Supplement: Supplementary file 1 — Supplementary Material 1. [file 12884_2026_9156_MOESM1_ESM.docx]

Table 1 Definitions of key variables used in the study

| **Variable** | **Definition** | **Data source** | **Notes** |
| --- | --- | --- | --- |
| Maternal age | Age at delivery (years) | Medical records | Categorized as <35 and ≥35 years old |
| Baseline BMI, kg/m^2^ | Recorded at the first antenatal visit between 8 and 12 weeks of gestation. | Medical records | —— |
| Gestational weight gain, kg | Calculated as the predelivery maternal weight minus the weight obtained at the first prenatal visit, measured in kilograms. | Medical records | —— |
| Assisted reproductive technology | Includes ovulation induction, intrauterine insemination, and in vitro fertilization with embryo transfer. | Medical records | —— |
| Nulliparity | No prior delivery ≥28 weeks | Medical records | —— |
| Congenital uterine anomalies | Includes uterine septum (complete and incomplete), unicornuate uterus, bicornuate uterus, and didelphys uterus. | Medical records | —— |
| Autoimmune diseases | Includes systemic lupus erythematosus (SLE), antiphospholipid syndrome, rheumatoid arthritis, Sjögren’s syndrome, Hashimoto’s thyroiditis, Graves’ disease, systemic sclerosis, and multiple sclerosis. | Medical records | —— |
| Gestational hyperglycemia | An umbrella term that encompasses pregestational diabetes mellitus and gestational diabetes mellitus | Medical records | —— |
| Anemia | Hemoglobin <110 g/L during pregnancy | Medical records | —— |
| Polyhydramnios | Amniotic fluid index (AFI) ≥ 25 cm or the maximum vertical pocket (MVP) ≥ 8 cm, assessed by ultrasound. | Medical records | —— |
| Oligohydramnios | AFI ≤ 5 cm or the MVP ≤ 2 cm, assessed by ultrasound. | Medical records | —— |
| Prelabor rupture of membranes | Rupture of membranes (water breaking) before labor begins. | Medical records | —— |
| Oxytocin induction | Use of oxytocin to stimulate uterine contractions and induce or augment labor. | Medical records | —— |
| Chorioamnionitis | Based on clinical criteria, including maternal fever exceeding 38°C accompanied by one or more of the following: maternal leukocytosis (white blood cell count ≥15×10⁹/L), fetal tachycardia (baseline fetal heart rate ≥160 bpm), or purulent cervical discharge. | Medical records | —— |
| Gestational age at delivery | Weeks at delivery | Delivery records | Divided into term (≥37 weeks) and preterm (<37 weeks). |
